# Subconjunctival Delivery of Sorafenib-Tosylate-Loaded Cubosomes for Facilitated Diabetic Retinopathy Treatment: Formulation Development, Evaluation, Pharmacokinetic and Pharmacodynamic (PKPD) Studies

**DOI:** 10.3390/pharmaceutics15102419

**Published:** 2023-10-04

**Authors:** Sharadha Madhusudhan, Naresh Vishal Gupta, Mohamed Rahamathulla, Saravana Babu Chidambaram, Riyaz Ali M. Osmani, Mohammed Ghazwani, Mohammed Muqtader Ahmed, Syeda Ayesha Farhana, Mohammed Y. Sarhan, Ahmed Hediyal Tousif

**Affiliations:** 1 Department of Pharmaceutics, JSS College of Pharmacy, JSS Academy of Higher Education & Research, Mysuru 570015, Karnataka, India; msharadha1996@gmail.com; 2 Department of Pharmaceutics, College of Pharmacy, King Khalid University, Abha 62529, Saudi Arabia; rahapharm@gmail.com (M.R.); myghazwani@kku.edu.sa (M.G.); 3 Department of Pharmacology, JSS College of Pharmacy, JSS Academy of Higher Education & Research, Mysuru 570015, Karnataka, India; saravanababu.c@jssuni.edu.in (S.B.C.); tousif.a.h7@gmail.com (A.H.T.); 4 Centre for Experimental Pharmacology & Research, Toxicology, Central Animal Facility, JSS Academy of Higher Education & Research, Mysuru 570015, Karnataka, India; 5 Cancer Research Unit, King Khalid University, Abha 62529, Saudi Arabia; 6 Department of Pharmaceutics, College of Pharmacy, Prince Sattam Bin Abdul Aziz University, Al Kharj 11942, Saudi Arabia; muqtadernano@gmail.com; 7 Department of Pharmaceutics, Unaizah College of Pharmacy, Qassim University, Unaizah 51911, Saudi Arabia; a.farhana@qu.edu.sa; 8 Department of Special Surgery, The Hashemite University, Zarqa 13133, Jordan; drsarhan@hu.edu.jo

**Keywords:** diabetic retinopathy, cubosome nanocarriers, sorafenib tosylate, subconjunctival injection, VEGF

## Abstract

Diabetic retinopathy (DR) is a microvascular complication associated with vascular endothelial growth factor (VEGF) overexpression. Therapeutic delivery to the retina is a challenging phenomenon due to ocular biological barriers. Sorafenib tosylate (ST) is a lipophilic drug with low molecular weight, making it ineffective at bypassing the blood–retinal barrier (BRB) to reach the target site. Cubosomes are potential nanocarriers for encapsulating and releasing such drugs in a sustained manner. The present research aimed to compare the effects of sorafenib-tosylate-loaded cubosome nanocarriers (ST-CUBs) and a sorafenib tosylate suspension (ST-Suspension) via subconjunctival route in an experimental DR model. In this research, ST-CUBs were prepared using the melt dispersion emulsification technique. The distribution of prepared nanoparticles into the posterior eye segments was studied with confocal microscopy. The ST-CUBs were introduced into rats’ left eye via subconjunctival injection (SCJ) and compared with ST-Suspension to estimate the single-dose pharmacokinetic profile. Streptozotocin (STZ)-induced diabetic albino rats were treated with ST-CUBs and ST-Suspension through the SCJ route once a week for 28 days to measure the inhibitory effect of ST on the diabetic retina using histopathology and immunohistochemistry (IHC) examinations. Confocal microscopy and pharmacokinetic studies showed an improved concentration of ST from ST-CUBs in the retina. In the DR model, ST-CUB treatment using the SCJ route exhibited decreased expression levels of VEGF, pro-inflammatory cytokines, and adhesion molecules compared to ST-Suspension. From the noted research findings, it was concluded that the CUBs potentially enhanced the ST bioavailability. The study outcomes established that the developed nanocarriers were ideal for delivering the ST-CUBs via the SCJ route to target the retina for facilitated DR management.

## 1. Introduction

Diabetic retinopathy (DR) is a retinal microvascular complication of uncontrolled diabetes, resulting in irreversible visual impairment and blindness. Pathological reports indicate structural and functional changes in retinal cells [1]. Global projections suggest that the number of DR cases will reach 191 million by 2030 [2]. The precise cause and pathogenic mechanism of DR remain undefined, but several hypotheses have been proposed. These include alterations in the polyol pathway, chronic inflammation, the diacylglycerol (DAG)/protein kinase C (PKC) pathway, accelerated formation of advanced glycation end products (AGEs), oxidative stress, activation of the renin–angiotensin system (RAS), the involvement of growth factors like VEGF, and hemodynamic shifts. All these mechanisms contribute to damage to the BRB and the development of neovascularization [3]. DR is categorized as proliferative and non-proliferative based on neovascularization. However, retinal detachment primarily occurs in proliferative DR due to the neovascularization on the posterior side of the vitreous and retina [4]. Clinically, even with controlled blood sugar and blood pressure, preventing DR progression remains challenging. 

In diabetes, elevated levels of the angiogenic factor (VEGF), pro-inflammatory cytokines (IL-6, IL-1β and TNF-α), and adhesion molecules (ICAM-1) are reported in the vitreous humor and retina, contributing to DR pathogenesis. VEGF, in particular, plays a pivotal role in DR. Clinical trials involving anti-VEGF agents such as bevacizumab, pegaptanib, ranibizumab, and aflibercept have shown promising results in treating DR [5]. However, their administration via intravitreal injection is invasive and associated with several complications in the management of progressive and chronic ocular disorders. These complications include the expansion of the avascular foveal zone, endophthalmitis, uveitis, temporary intraocular pressure (IOP), increase, retinal detachment, cataracts, and macular hole development [6]. 

Sorafenib tosylate (ST) is a small molecule with a molecular weight of 637 Da, and it functions as a type II multi-targeted tyrosine kinase inhibitor that specifically targets VEGFR-2 [7]. In 2005, the FDA granted its initial approval for the treatment of thyroid, renal cell, and hepatocellular carcinoma, with Bayer HealthCare Pharmaceuticals Inc., Wayne, NJ, USA, as the manufacturer. 

Recent findings have showcased its beneficial effects in treating retinal neovascular pathologies, including age-related macular degeneration (AMD), diabetic retinopathy, and choroidal neovascularization [8,9,10]. Kernt et al. (2011) reported the potential of ST in reducing the expression of VEGFR-1 and VEGFR-2 on human RPE cells during angiogenic treatments [11]. Numerous drug delivery systems have been developed for ST, such as microparticles [12], microemulsions [13], liposomes [14], nanoparticles [15,16], and more, to facilitate drug delivery to the posterior eye segments. Among these emerging approaches, it is very likely that nanoparticles are promising strategies that can enhance a drug’s half-life (t_½_), increase maximum drug concentration (C_max_), elevate drug concentration in target tissues by enhancing bioavailability, reduce dosing frequency and toxicity, and achieve sustained, prolonged drug release [17,18].

One of the versatile lipid-based nanocarrier systems is liquid crystalline nanoparticles (LCNPs), often referred to as “cubosomes” due to their cubic phase structure. LCNPs outshine other lipid-based and polymeric nanoparticles due to their high surface area, self-assembling capacity, formulation of a sustained drug release matrix, thermal stability, and excellent encapsulation efficiency [19,20]. Recently, LCNPs have gained significant attention for effective ophthalmic drug delivery owing to their high ocular bioavailability [21], prolonged retention time [22,23,24], minimal ocular irritation [25], and sustained drug release [26]. Given these features, LCNPs are regarded as an excellent vehicle for ocular drug delivery. 

The present study aimed to explore the potential of ST-loaded nanocrystalline cubosomes by investigating the expression of VEGF and pro-inflammatory factors in an STZ-induced DR rat model. 

## 2. Materials and Methods

### 2.1. Chemicals

Sorafenib tosylate (ST) was generously provided by Dr. Krishna Bhavanasi at Natco Pharma Ltd. (Kothur, Telangana, India). Monoolein (MO) (RYLO MG 19 Pharma) was sourced from Danisco Ingredients (Grinsted, Denmark). Pluronic F-127, Rhodamine B, and Streptozotocin (CAS ID 18883-66-4) were purchased from Sigma-Aldrich (Bangalore, India). Liquid chromatography–mass spectroscopy (LCMS)-grade solvents, hematoxylin, and eosin stain were obtained from Merck (Bangalore, India). Antibodies were procured from Santa Cruz Biotechnology, Inc. (Santa Cruz, CA, USA), and Abcam (Cambridge, MA, USA). All other chemicals utilized were of analytical reagent grade.

### 2.2. Preparation, Characterization, and Storage Stability Studies of Cubosome Nanocarriers

#### 2.2.1. Preparation of Cubosome Nanocarriers

Sorafenib-tosylate-loaded cubic nanocarriers (ST-CUBs) were prepared using a hot melt dispersion emulsification technique as described by Eldeeb et al. (2019) with some modifications. In brief, MO at 5.0% (*w*/*v*) and Pluronic F-127 at 0.65% (*w*/*v*) were melted to 70 °C, followed by the addition of ST (0.3%) to the molten lipid phase. This mixture was then dropped to an aqueous phase at 94.35% (*w*/*v*), containing water heated to the same temperature. Then, the dispersion was emulsified using a high-speed homogenizer (IKA T-25 digital ULTRA-TURRAX^®^, Bengaluru, India) at 10,000 rpm for 10 min and stirred on a magnetic stirrer (Remi 1 MLH) for 30 min at 1500 rpm until it cooled completely [27]. 

#### 2.2.2. Characterization of Cubosome Nanocarriers

##### Particle Size, Polydispersity Index (PDI), and Zeta Potential (ZP)

The particle size, polydispersity index (PDI), and zeta potential (ζ) were determined using the dynamic light scattering (DLS) method (Zetasizer, Nano ZS90, Malvern, UK). The developed formulation was appropriately diluted to 1:100 in Milli-Q water and transferred to a polystyrene cuvette with a 10 mm path length at 25 °C, with a light scattering angle of 90°. The average particle diameter and width of the diameter were reported as particle size and PDI. The ζ of the developed formulation was assessed using disposable zeta cells with water as the dispersant.

##### HR-TEM (High Resolution-Transmission Electron Microscopy)

The morphological features of the formulated ST-CUBs were imaged using HR-TEM. A microliter droplet of ST-CUBs was diluted in distilled water (1:100) and pipetted onto a carbon-coated TEM grid with a 200-mesh size. It was then negatively stained with 2% phosphotungstic acid and allowed to settle for half a minute. The drop was manually blotted with an absorbent filter paper to forma thin film. The grid was then transferred to HR-TEM (JEOL, JM 2100, Tokyo, Japan). Images were digitally captured in accelerating voltage imaging mode (200 kV) with a camera length and resolution of 499.606 mm and 0.24 nm, respectively, at a scale of 20 nm (×60 k) [28]. 

##### Drug Encapsulation Efficiency

The amount of ST encapsulated in cubic nanocarriers was estimated using a centrifugation method. Cubic nanocarriers were centrifuged at 5000 rpm for 30 min at 4 °C (Micro-centrifuge, Remi, RM-12C DX). The clear supernatant was collected and analyzed for ST using an ultraperformance liquid chromatography–mass spectrometry (UPLC-MS) system. The percentage encapsulation efficiency (% EE) was calculated using the following Equation (1):

(1) $$\%\;\mathrm{EE}=\frac{\left(total\;amount\;of\;ST\;used-free\;ST\;in\;supernatant\right)}{total\;amount\;of\;ST\;used}\times 100$$

#### 2.2.3. Storage Stability Studies of Cubosome Nanocarriers

The stability of freshly prepared ST-CUBs was assessed over 90 days at two different temperatures: 25 °C/60% relative humidity (RH) (room temperature—long-term condition and 2–8 °C (refrigerated condition), in accordance with ICH guidelines. Samples were withdrawn at intervals of 0, 15, 30, 45, 60, 75, and 90 days and characterized for changes in globule size, polydispersity index (PDI), and zeta potential using the DLS method (Zetasizer, Nano ZS90, Malvern, UK), EE % using RP-HPLC (Shimadzu, LC-2030C Plus i-SERIES, Kyoto, Japan), and organoleptic features (color, odor, aggregation, and precipitation) [29].

### 2.3. In Vivo Studies

#### 2.3.1. Animals and Husbandry Conditions

The experimental protocol received approval from the Institutional Animal Ethics Committee (JSSAHER/CPT/IAEC/066/2021) of JSS AHER, Mysuru. The research procedures adhered to the Association for Research in Vision and Ophthalmology (ARVO) Statement for the Use of Animals in Ophthalmic and Vision Research. Female Wistar Albino rats, weighing 200–250 g (eight to ten weeks old), were procured from Aditya Biosys, Bangalore, and utilized in this study. All animals underwent a seven-day acclimatization period before the experiments and were housed under standard laboratory conditions, which included a temperature of 22 ± 3 °C, relative humidity between 45 and 65%, and a 12-hour light–dark cycle throughout the study. Standard rodent chow and purified water were available ad libitum. The rats were housed in clean and sterilized polycarbonate rat cages with solid floors covered with corncobs as bedding material. 

#### 2.3.2. In Vivo Confocal Laser Scanning Microscopy (CLSM) Imaging Test

The distribution of nanoparticles following periocular injection (subconjunctival route) was studied using CLSM. Rhodamine B (RhB), a water-soluble fluorescent dye (0.03%), was used for the CLSM study. Six female albino rats were divided into two groups, each containing three rats. Group I received 50 µL of RhB dye loaded in ST-CUBs through a topical route using an eye dropper; Group II received 50 µL of RhB dye loaded in ST-CUBs through a subconjunctival route using a 27-gauge needle. After 48 hours, the animals were euthanized, their eyes were enucleated, and the retinas were cross-sectioned from the whole eyeball for examination using CLSM (Stellaris 5, Leica Microsystems, Mannheim, Germany) with a Power HyD S detector after excitation at 488 nm and emission at 560 nm. All images were captured at 40× magnification using LAS X software 3.3 [30].

#### 2.3.3. Single-Dose Pharmacokinetic and Biodistribution Studies 

For pharmacokinetic studies, rats were divided into three groups, each containing 18 rats. The left eye (ipsilateral) of each rat received the specified treatment as outlined in Table 1. The right eye (contralateral) was treated similarly with 0.1 mL of normal saline. 

Before SCJ injection, rats were anesthetized using a mixture of ketamine (Aneket^®^, Neon Laboratories Limited, New Delhi, Delhi, India) and xylazine (Xylaxin^®^, Indian Immunologicals Limited, New Delhi, Delhi, India) in a 2:1 ratio, administered intramuscularly (IM) at a dose of 0.6 mL/kg. Further local anesthesia was induced by instilling 0.5% proparacaine hydrochloride eye drops into the rat’s eyes. Subsequently, the rats received 50 µL of the respective treatment in the subconjunctival space of the left eye. Any slight hemorrhage post-injection was managed by applying mild pressure with cotton-swab applicators. The dose of ST in the formulation (ST-CUBs) and suspension was 150 µg/50 µL. Blood samples and posterior ocular tissue samples (vitreous humor and retina) were collected at predetermined time points, specifically 2, 4, 6, 8, 12, and 24 h following SCJ injection. At each time point, the left eye of the rats was enucleated and immediately frozen at −80°C in an ultra-low-temperature freezer (u725-Innova, Eppendorf, New Brunswick Scientific, Germany). The concentration of ST in plasma and ocular tissue samples was evaluated by UPLC-MS in the respective samples. The tissue samples were processed as described below.

##### Preparation and Determination of Plasma Drug Concentration

At predetermined time points, the blood samples were collected into EDTA-coated tubes through retro-orbital puncture using sterile hematocrit capillary tubes. Plasma was separated by centrifugation (REMI, Mumbai, Maharashtra, India) at 3500 rpm for 10 minutes at room temperature. The separated plasma was sonicated (Microclean-101, Oscar Make, Mumbai, Maharashtra, India) with 2 mL of methanol for 30 min. Then, the solution was centrifuged at 10,000 rpm for 10 min at ambient temperature to precipitate proteins. The supernatant was filtered through a 0.22 µm nylon filter, and 5 µL of the filtered supernatant was injected into the UPLC-MS system [31].

##### Preparation and Determination of Vitreous Humor Concentration

Similarly, vitreous humor was collected at each time point with a glass capillary through a hole made with a 20G needle in the posterior eye segment of the rat. The weighed vitreous humor was mixed with 2 mL of methanol and sonicated using an ultrasonic bath (Microclean-101, Oscar Make) for 30 min. Then, the solution was centrifuged at 10,000 rpm for 10 min at ambient temperature to precipitate proteins. The supernatant obtained was filtered through a 0.22 µm nylon filter, and 5 µL of the filtered supernatant was injected into the UPLC-MS system [32].

##### Preparation and Evaluation of Retina Concentration

The retina, detached from the choroid and the optic nerve, was weighed and cut into smaller pieces in a frozen condition and transferred to 10 mL of PBS for homogenization (FastPrep-24^TM^ Classic high-speed homogenizer). The homogenate was then mixed with methanol and centrifuged at 10,000 rpm for 10 min at 4 °C to precipitate proteins. The supernatant was collected to estimate the ST content by UPLC-MS [33].

##### UPLC-MS Specification and Mobile Phase

The clear supernatant collected from rat plasma and ocular tissues was injected (5 µL) into a UPLC-MS system (Acquity H-class, Waters Corporation, Milford, MA, USA) equipped with an integrated vacuum degasser and Ultra-performance binary solvent manager (Serial # C10UPA554M, Waters Corporation, Singapore).

Chromatography Conditions: A C18 Column (Acquity UPLC BEH^®^ 1.7 µM, 2.1 × 50 mm) was used at ambient temperature. The mobile phase system comprised 0.1% formic acid in water (A) and acetonitrile (B) at a flow rate of 0.6 mL/min. The injection volume was 5 µL. A gradient elution procedure was followed: from the initial to 4 min (changing from 10 to 95% B), held for 4 min, from 8 to 8.1 min (changing from 90 to10% B), and held at 10% for 1.9 min. 

Mass Spectrometry Conditions: The mass spectrometry was conducted in positive polarity electrospray combined ionization (ESCi) source mode. Acquisition parameters included a 50 L/h cone gas (nitrogen) flow, 750 L/h desolvation gas (nitrogen) flow, 450 °C probe temperature, 30 V sampling cone voltage, 150 °C source temperature, 80 V source offset voltage, sample infusion flow rate of 5 mL/min, and collision energy ramp varying from 6 eV (argon, collision gas). The mass range covered was from 50 to 1500 *m*/*z*. All data were acquired and processed using Waters Corporation’s Mass Lynx software (V4.1 Milford, MA, USA).

##### Detection of Pharmacokinetic Parameters

Pharmacokinetic (PK) parameters, such as C_max_ (ng/mL), T_max_ (h), K_el_ (h^−1^), T_1/2_ (h), AUC_(0–24)_ (ng·h/mL), AUC_(24–∞)_ (ng·h/mL), AUC_(0–∞)_ (ng·h/mL), AUMC_(0–24)_ (ng·h^2^/mL), AUMC_(24–∞)_ (ng·h^2^/mL), AUMC_(0–∞)_ (ng·h^2^/mL), and MRT_(0–∞)_ (h), were calculated by considering a one-compartment model. The % relative bioavailability (RB) in the retina was calculated for the formulation and suspension after SCJ injection using Equation (2):

(2) $$Relative\;Bioavailabilty=\frac{\left[AUC(0-24)\;of\;ST-CUBs\right]}{\left[AUC(0-24)\;of\;ST-Suspension\right]}\times 100$$

Graphs were plotted for plasma, vitreous, and retina ST concentration vs. time between ST-CUBs and the ST-Suspension groups. Statistical analysis was performed using GraphPad Prism software (version 9.4.1).

#### 2.3.4. In Vivo Safety Assessment

The safety of the formulated ST-loaded cubic nanocarriers was evaluated after SCJ injection through visual inspection and retinal histopathology. The subconjunctival area of the rat eye was visually inspected for tissue inflammation and edema twice, on the 1st and 7th days of the observation period. For the histopathology study, rats were euthanized after 7 days. The eyes were enucleated, and retinal tissue was blocked in paraffin, sectioned, stained with H&E, and viewed at 400× magnification with a research trinocular research microscope (Lx-500 LED, Labomed, Los Angeles, CA, USA). Images were captured using a MiaCam USB 3.0 CMOS Camera AR 6 Pro (M/S Lab Systems, Bengaluru, India) microscope camera attached to Image AR Pro software version 2.0 [30,34].

#### 2.3.5. Pharmacodynamic Studies

##### Groupings and Treatment

Diabetes was induced in experimental rats using streptozotocin (STZ). In overnight-fasted rats, STZ (60 mg/kg, i.p) freshly prepared in a 10 mM cold citrate buffer (pH 4.5)) was injected. Non-diabetic rats received an equal volume of vehicle (10 mM cold citrate buffer, pH 4.5) [35,36]. Seventy-two hours after STZ injection, rats with blood glucose levels ≥250 mg/mL were selected for the study. Animals were randomized into 4 groups (n = 6/group): GN: non-diabetic rats; GD: rats in which diabetes was induced (Positive Control); GDSS: rats in which diabetes was induced, treated with ST-Suspension; GDSC: rats in which diabetes was induced, treated with ST-CUBs.

##### Body Weight, Blood Glucose, and Glycosylated Hemoglobin (HbA1c) Determination

Throughout the study period, body weight, plasma glucose, and glycosylated hemoglobin (HbA1c) levels were measured weekly. Blood glucose levels were measured using a digital glucometer (Morepen GlucoOne, BG-03) and glucose test strips. HbA1c was determined using the Turbodyne^TM^HbA1c immunoturbidimetric assay.

##### Assessment of Retinal Morphology by Hematoxylin and Eosin (H&E) Staining

Both the non-diabetic rats and diabetic rats were observed for retinal morphological changes following the induction of diabetes for the 1st and 2nd months. The rats were euthanized, and their eyeballs were enucleated and rinsed with ice-cold normal saline. The retina was isolated and immersion-fixed in 10% buffered formalin upon removal and then blocked in paraffin blocks. Thin sections (4 μm) of the tissue block were cut using a microtome. The sectioned retinal tissue was deparaffinized, dehydrated, stained with hematoxylin and eosin (H&E), washed, and sealed with neutral resin. Subsequently, images were captured at 400× magnification using a MiaCam CMOS AR 6pro microscope camera linked to Image AR Pro software.

Microstructural retinal changes were observed by blinded observers and scored from 0 to 4 based on histopathologic aspects (0 = absence of lesion; 1 = fewer lesions; 2 = mild lesions; 3 = moderate lesions; and 4 = severe lesions).

##### Treatment Protocol in a Rat Model of Retinopathy

The vehicle or test drug was instilled in the left eye of the respective groups once a week for 28 days. ST was injected at a dose of 150 µg/50 µL. Rats were subjected to histopathological examination, and the protein expression of VEGF, cytokines, and adhesion molecules was analyzed using immunohistochemistry.

##### Immunohistochemistry (VEGF, Pro-Inflammatory Cytokines, and Adhesion Molecules)

The expression of VEGF, IL-1β, IL-6, TNF-α, and ICAM-1 was examined in rat retinal tissue. This was carried out after performing paraffin sectioning as explained in the above H&E section. The retinal sections on the slides were dewaxed, washed with water and phosphate-buffered saline (PBS), and covered with 3% hydrogen peroxide to block peroxidase. The slides were then rinsed thrice with water and PBS, heated with unmasking reagents to retrieve antigens, and then dried. The sections were blocked using 0.25% bovine serum albumin (BSA) for 30 min at 37 °C. Then, BSA was wiped, and sections were incubated at 4 °C overnight with a primary antibody at the appropriate dilution: 1:300 VEGF, 1:50 IL-6, 1:100 IL-1β, 1:100 TNF-α, and 1:500 ICAM-1. The sections were rinsed thrice with PBS and covered with appropriate secondary antibodies (Rabbit Anti-Rat IgG, HRP) for 30 min. The slides were again washed thrice with PBS and stained with 3,3′-Diaminobenzidine (DAB) substrate buffer using a DAB chromogenic kit for 5 min. The slides were washed with running tap water and counterstained with hematoxylin for 3 min. The slides were differentiated with 1% acid alcohol after washing with tap water. The slides were then dehydrated using 80%, 90%, and 100% alcohol, cleared with three changes of xylene, and mounted with dibutyl phthalate polystyrene xylene (DPX). Finally, the images were obtained under a ZEISS Axioplan 2 microscope connected to Multi-spectrum Image analysis software [37].

The scoring for the expression levels of VEGF, IL-6, IL-1β, TNF-α, and ICAM-1in different layers of the retina was performed by a blinded observer and categorized as follows: 0 (no expression), 1 (normal expression/<25% of marker expression in the retina), 2 (mild expression/25–50% of marker expression in the retina), 3 (moderate expression/50–75% of marker expression in the retina), and 4 (severe expression/>75% of marker expression in the retina).

### 2.4. Statistics

Statistical analysis was performed using GraphPad Prism software (version 9.4.1). Data were expressed as mean ± SD. Body weight, plasma glucose level, HbA1c level, and UPLC-MS data were analyzed using an unpaired *t*-test. Immunohistological analysis was performed using one-way ANOVA followed by Tukey’s multiple comparison test. A *p* value of 0.05 was generally considered statistically significant (* significant (*p* ≤ 0.05), ** very significant (*p* ≤ 0.01), *** (*p* ≤ 0.001), and **** (*p* ≤ 0.0001) extremely significant).

## 3. Results

### 3.1. Characterization and Storage Stability of Sorafenib-Tosylate-Loaded Cubosome Nanocarriers

**Figure 1:**
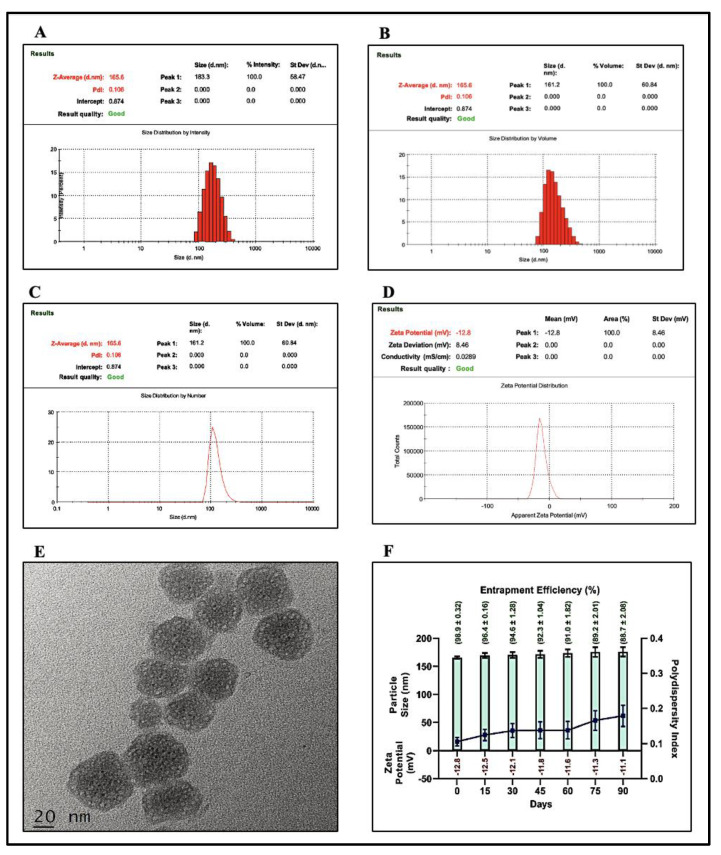
ST-CUB characterization and stability studies. (**A**) Histogram of size distribution by intensity;(**B**) histogram of size distribution by volume; (**C**) size distribution by number; (**D**) zeta potential; (**E**) HR-TEM image; (**F**) particle size, PDI, zeta potential and % entrapment efficiency following storage stability at 25 °C/60% RH.

The Z-average size of the developed ST-CUBs, as determined by intensity mean (Figure 1A), volume mean (Figure 1B), and number mean (Figure 1C) measurements, was 165.6 nm. This size distribution was further characterized by specific percentiles, with D10 at 114 nm, D50 at 174 nm, and D90 at 270 nm. The PDI was measured at 0.106, indicating a relatively uniform particle size distribution. The zeta potential of ST-CUBs was measured at −12.8 mV, indicating the stability of the cubosome nanocarrier system (Figure 1D). HR-TEM images clearly delineated predominantly square liquid crystalline morphologies of ST-CUBs at the nanoscale without clumpy aggregation (Figure 1E). Additionally, the % EE was found to be high, specifically at 97.26 ± 2.18%.

Long-term stability studies are crucial when investigating lipid-based nanocarriers. Over a period of 3 months, the organoleptic features of the ST-CUB dispersion stored at room temperature remained unchanged. As depicted in Figure 1F, ST-CUBs stored at 25 °C exhibited an insignificant increase in particle size from 165.6 ± 2.1 nm to 176.0 ± 8.5 nm, PDI increased from 0.106 ± 0.01 to 0.179 ± 0.03, zeta potential decreased from −12.8 to −11.1, and % EE decreased from 98.9 ± 0.32% to 88.7 ± 2.08% over 90 days. Conversely, it was noticed that ST-CUBs stored at 2–8°C exhibited a phase separation of the nano dispersion over a month. This circumstance is associated with accelerated crystallization of the colloids dispersed in GMO on cooling to a controlled temperature (2–8 °C) from room temperature. Thus, it can be concluded that ST-CUBs were less pronounced at 25 °C.

### 3.2. In Vivo CLSM Imaging Study

**Figure 2:**
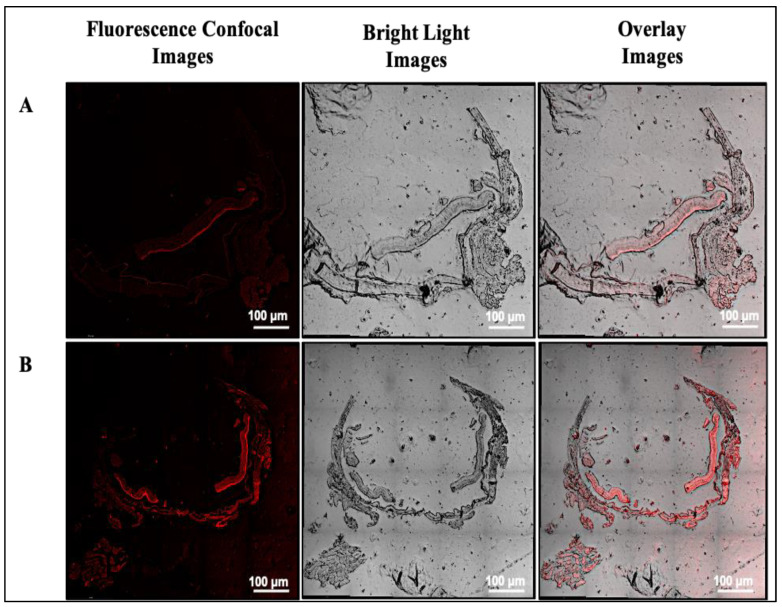
Confocal microscopy images (fluorescent/dark field—left; bright field—middle; overlay image—right) of sectioned retina from rat eyeball after treatment with RhB-loaded ST-CUBs administered via (**A**) topical route and (**B**) SCJ route. The scale bars are 100 μm.

Figure 2A and B show CLSM images of sectioned rat eyes after various treatments. The confocal and overlay images confirm the presence of red fluorescence indicating the distribution of the RhB dye in the posterior eye segment. As visualized in the confocal image, topical administration of RhB-loaded ST-CUBs shows low fluorescence intensity (low distribution) (Figure 2A), whereas SCJ administration of RhB-loaded ST-CUBs in the retina/choroid and sclera showed high fluorescence intensity (high distribution) (Figure 2B).

### 3.3. UPLC-MS Linearity

**Figure 3:**
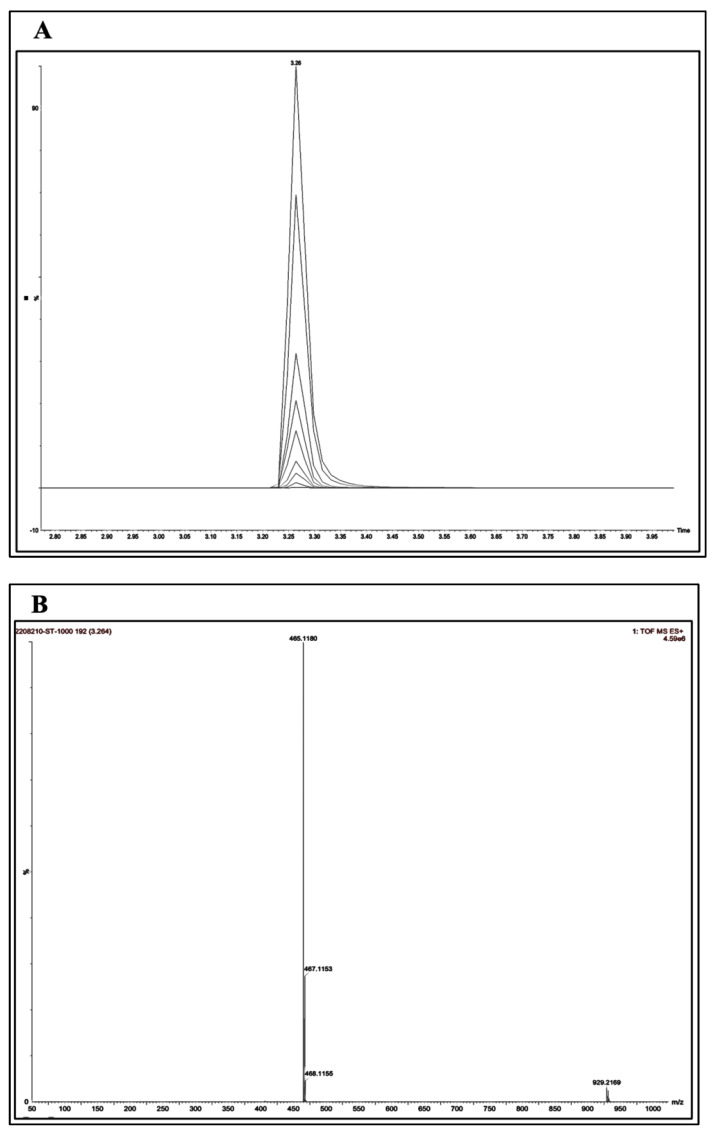

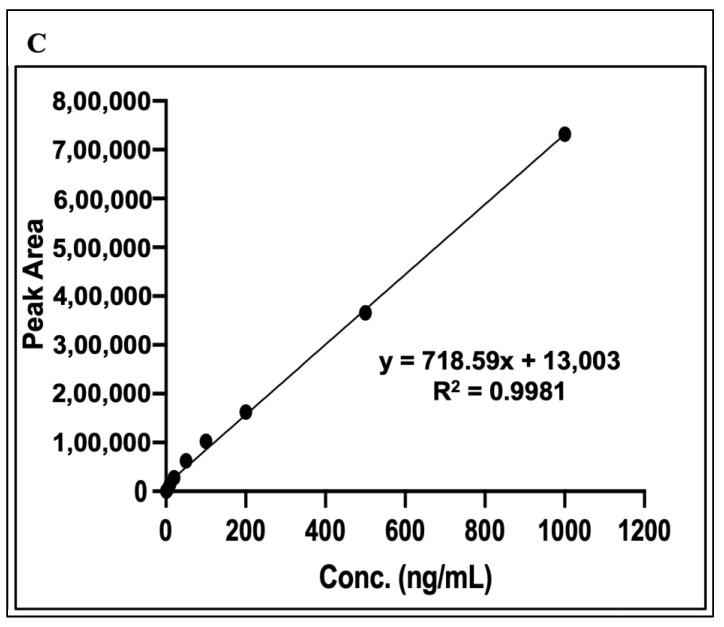
ST chromatogram obtained from UPLC-MS, (**A**) overlay of the retention time (3.26 min), (**B**) positive ion chromatogram (*m*/*z* = 465.1), and (**C**) calibration curve of ST with nine standard points in triplicates.

The ST concentration was determined in the plasma and ocular tissues using the UPLC-MS. The retention time was found to be approximately 3.26 min (Figure 3A), with a good resolution peak. Mass-selective detection (MSD) was conducted in the positive ion mode with selected-ion monitoring (SIM) at 465.1 for ST (*m*/*z*) (Figure 3B). The linear calibration curves were obtained by plotting the peak area of chromatograms (standard concentration) versus particular concentration (1 ng/mL, 5 ng/mL, 10 ng/mL, 20 ng/mL, 50 ng/mL, 100 ng/mL, 200 ng/mL, 500 ng/mL, and 1000 ng/mL) (Figure 3C). The R^2^ was 0.9981, with an equation of y = 718.59x + 13,003. The unknown sample concentration of the plasma and ocular samples was measured by interpolating from the calibration curve.

#### Single-Dose Pharmacokinetic and Biodistribution Study

**Figure 4:**
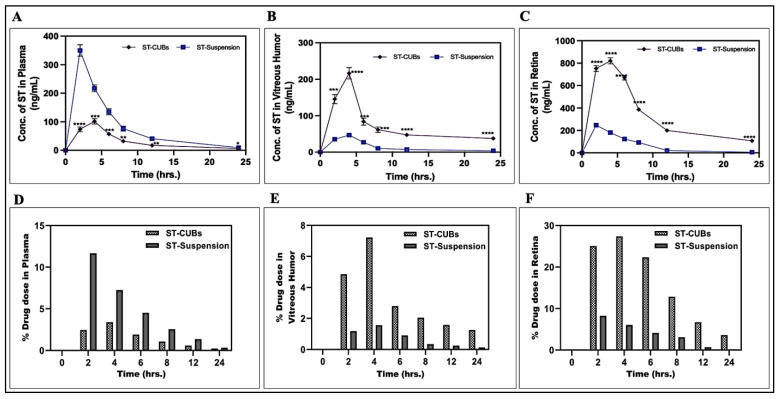
ST concentration–time profile and percentage of ST dose profile of ST-CUBs and ST-Suspension groups following SCJ route to albino rats at 2 h, 4 h, 6 h, 8 h, 12 h, and 24 h. (**A**) Plasma drug profile, (**B**) vitreous humor drug profile, (**C**) retina drug profile, (**D**) % ST dose profile in plasma, (**E**) % ST dose profile in vitreous humor, and (**F**) % ST dose profile in retina. Each data point is expressed as mean ± SD (*n* = 3 at each time point in each group). ST-CUBs were significantly different from ST-Suspension at * *p* ≤ 0.05, ** *p* ≤0.01, *** *p* ≤0.001, and **** *p* ≤ 0.0001.

The dose of ST injected was kept constant for better comparison. Following a single subconjunctival injection (150 µg/50 µL), the ST concentrations obtained in plasma, vitreous humor, and retina from the two groups were compared after respective treatments as detailed in Figure 4. The PK data of the ST-CUBs group reflected the ST released from CUBs, and the PK data of the ST-Suspension group reflected the ST (free drug/unbounded drug/unloaded drug) available in the biological samples.

The ST concentration obtained in plasma after SCJ injection of ST-Suspension was noticeably higher than that of ST-CUBs, as depicted in the relative line diagram (Figure 4A). In addition, the ST concentration obtained in vitreous humor and retina after SCJ injection of ST-CUBs was significantly higher than that of SCJ injection of ST-Suspension (Figure 4B and Figure 4C). The percent of the drug dose from ST-CUBs was found to be higher in the retina (Figure 4F) and vitreous humor (Figure 4E), whereas the percent of the drug dose from ST-Suspension was higher in plasma (Figure 4D) at various time intervals.

The pharmacokinetic parameters of ST obtained in plasma, vitreous humor, and retina are reported in Table 2. The p value of< 0.0001 from the two-tailed *t*-test showed statistically significant differences in the PK parameters obtained for ST in the retina after SCJ injection of ST-CUBs.

In the current study, the ST concentration in the retina was determined after SCJ administration of ST-CUBs and ST-Suspension. C_max_ after SCJ injection of ST-CUBs and ST-Suspension was 820.75 ± 37.41 ng and 246.94 ± 43.14 ng, respectively (*p* < 0.0001). T_max_ for ST-CUBs and ST-Suspension was found to be 4 h and 2 h, respectively. The elimination rate constant (K_el_) and half-life (T_1/2_) of ST-CUBs were 0.0420 ± 0.004 h^−1^and 16.4756 ± 0.215 h, whereas for ST-Suspension, they were 0.0561 ± 0.06 h^−1^and 12.338 ± 0.283 h, respectively. Furthermore, there was a 5.02-fold increase in AUC_0–24_, a 6.21-fold increase in AUC_0–∞_, a 6.78-fold increase in AUMC_0–24_, a 13.41-fold increase in AUMC_0–∞_, and a 2.1-fold increase in MRT_0–∞_ for ST-CUBs compared with ST-Suspension. Further, the relative bioavailability of ST in the retina was found to be 502.11 ± 10.24%.

### 3.4. In Vivo Safety Assessment

**Figure 5:**
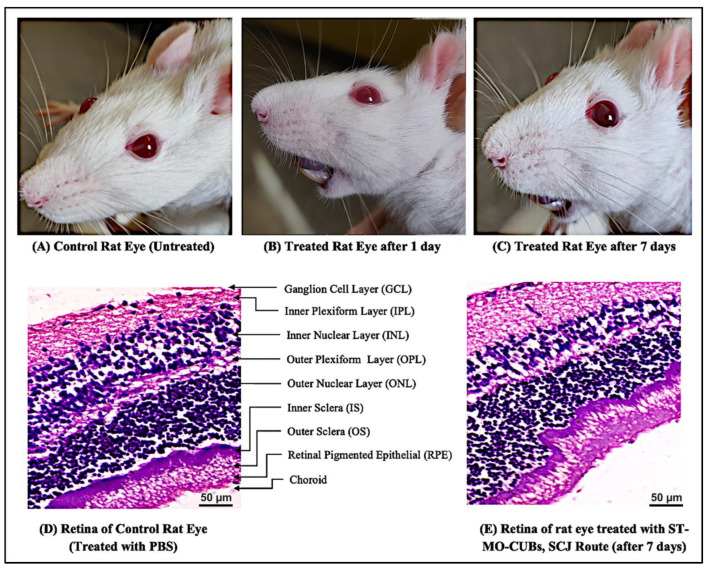
(**A**) Visual observation of untreated rat’s left eye. (**B**) Visual observation of treated rat’s left eye with ST-CUBs, SCJ route, after 1 day. (**C**) Visual observation of treated rat’s left eye with ST-CUBs, SCJ route, after 7 days. (**D**) H&E histological evaluation of retina of untreated rat’s left eye at 400×. (**E**) H&E histological evaluation of retina of treated rat’s left eye with ST-CUBs, SCJ route, after 7 days at 400×.

The gross pathological observations of the treatment-group rat eye on day 1 (Figure 5B) and day 7 (Figure 5C) appear similar to those of the control rat eye (Figure 5A). Particularly, there were no signs of edema, inflammation, or injury surrounding the injected sites.

The histopathological changes in the rat retina were assessed after 7 days of treatment and compared with those of the control group. H&E staining revealed no microstructural injury, necrosis, or granuloma in the injected eyes of the ST-CUB-treated group (Figure 5E) and appeared similar to that of the control group (Figure 5D), which supports the gross pathology examination data.

### 3.5. Pharmacodynamic Study

#### 3.5.1. Physiological State of Rats

Non-diabetic rats appeared clean with normal fur and exhibited typical feeding and water intake behavior. Vehicle-treated diabetic rats were observed to have opaque eyeballs due to vascular alterations in the retinal framework following the induction of hyperglycemia. These animals experienced weight loss, a dull coat and fur shedding, polyphagia, polydipsia, and polyuria.

#### 3.5.2. Body Weight, Fasting Blood Glucose, and Glycosylated Hemoglobin (HbA1c) Determination in the Rats

**Figure 6:**
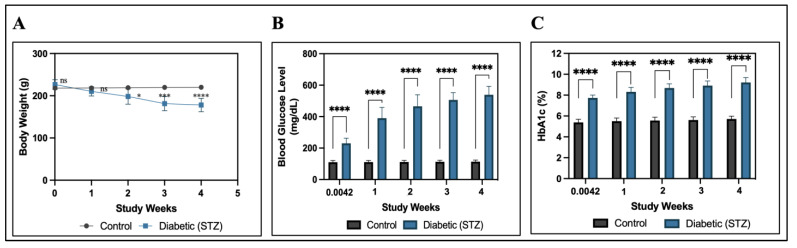
Graphs confirming the development of the diabetes rat model. (**A**) Body weight, (**B**) blood glucose level, and (**C**) HbA1c levels from control and diabetic rats during 4 weeks after STZ induction. The body weight of STZ-induced diabetic rats was significantly lower than that of the control rats, whereas the blood glucose level and HbA1c level of STZ-induced diabetic rats were significantly higher than those of the control rats at * *p* ≤ 0.05, *** *p* ≤ 0.001, and **** *p* ≤ 0.0001. ^ns^ represents non-significant at *p* > 0.05. Data represented as mean ± SD (*n* = 6 in each group).

The body weight, blood glucose, and HbA1c levels of control (CG) and STZ-treated rats were measured before and after the development of diabetes at 72 h and on the 1st, 2nd, 3rd, and 4th weeks, and data are shown in Figure 6. The body weight of control rats continuously increased throughout the study, from 218 ± 3.42 g to 220.16 ± 1.07 g, whereas the body weight of STZ-induced rats decreased from 226.50 ± 11.32 g to 178.00 ± 15.91 g, and a gradual decrease was observed from the 2nd week. Furthermore, the fasting blood glucose level (mg/dL) of normoglycemic control rats remained normal throughout the study, ranging from 110.16 ± 10.53 mg/dL to 113.83 ± 9.70 mg/dL, and varied within a narrow range (≤125 mg/dL), whereas STZ-induced rats showed a significant increase after 72 h of STZ induction, from 230.50 ± 32.43 mg/dL to 539.33 ± 53.96 mg/dL, with a sharp elevation (>250 mg/dL) observed from the 1st week to the 4th week. The level of HbA1c (%) was found to be higher in STZ-treated rats, ranging from 7.73 ± 0.27% to 9.21 ± 0.47%, and elevated significantly (>8%) compared to control rats, whoseHbA1c levels ranged from 5.38 ± 0.30% to 5.71 ± 0.27% (<6%). This confirms the depiction of diabetes.

#### 3.5.3. Retinal Morphology by H&E Staining

**Figure 7:**
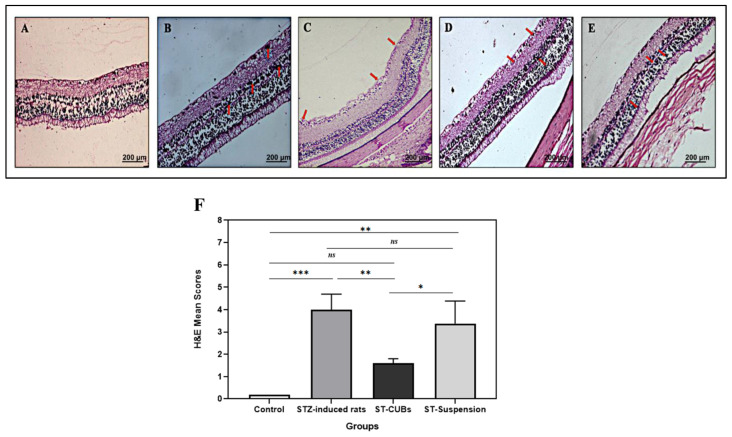
The H&E-stained retinal sections of (**A**) control group, (**B**) diabetic group after 1 month of STZ induction, (**C**) diabetic group after 2 months of STZ induction, (**D**) ST-CUB treatment for 28 days in diabetic group after 2 months of STZ induction, (**E**) ST-Suspension treatment for 28 days in diabetic group after 2 months of STZ induction. (**F**) Unbiased mean scores on H&E histopathological sections at 200× by blinded spectators, where 0 signifies absence of lesion and 4 signifies severe lesions. The SCJ-administered ST-CUBs exhibited significant recovery from DR pathological vascular alterations compared to the ST-Suspension group. Red arrows indicate the vascular alterations in the retinal structure. ^ns^ represents non-significant at *p* > 0.05, * *p* ≤ 0.05, ** *p* ≤ 0.01, and *** *p* ≤ 0.001. Data represented as mean ± SD (*n* = 6 in each group).

From the H&E-stained sections, the retinas of control rats were found to be clear, complete, evenly, and densely distributed, with smooth retinal surfaces, normal retinal thickness (inner plexiform layer (IPL), inner nuclear layer (INL), outer nuclear layer (ONL)), and intact retinal layers. Additionally, the inner and outer segments of photoreceptor nuclei and retinal ganglion cells were highly organized, with normal capillaries (Figure 7). 

In contrast, mild changes were observed in a structural layer of the retina after 1 month of STZ treatment. Multifocal apoptosis of neuronal cells was observed in the INL, ONL, and ganglion cell layer (GCL) in the retina (Figure 7B). Moderate changes were observed after 2 months, as depicted in Figure 7C. The changes in diabetic rats were unclear, incomplete, uneven, and loosely distributed, with thin and impaired retinal layers. The thickness of INL and ONL, along with the number of ganglion cells, was found to be decreased. The inner and outer segments of photoreceptor nuclei showed moderate disorganization, and vacuoles were noticeable. The blood vessels were found to be dilated/vasodilated and filled with interstitial edema fluid. The retinal lesions were graded from 0 to 4 (no, minimal, mild, moderate, and severe lesions) (mean ± SD, *n* = 6). The total grade obtained for retinal lesions in STZ-induced diabetic rats was 3.99 ± 0.7. All these pathological vascular alterations were noticed in the STZ-induced rats after 2 months. Hence, the DR model was successful in STZ-induced diabetic rats.

### 3.6. Treatment of Retinopathy

#### 3.6.1. ST-CUBs Markedly Prevented Changes in the Retinal Morphology of STZ-Diabetic Rats

Treatment with ST-loaded cubic nanocarriers and ST-Suspension partially restored the retinal morphology when compared to the diabetic group. There was moderate mitigation in retinal structure in the ST-CUB-treated group compared to that in the ST-Suspension group. After treatment with ST-CUBs, the retinal structure appeared fairly arranged, increased in thickness (INL and ONL) and retinal ganglion cells, and exhibited a noticeable partial organization of photoreceptor nuclei. Mild degeneration and apoptosis of ganglion cells in the GCL and vasoconstriction with mild edema were also observed (Figure 7D). After treatment with ST-Suspension, moderate degeneration was noticed in the INL and INL of the retina (Figure 7E). The total grade obtained for the reduction in retinal lesions for the groups treated with ST-CUBs and ST-Suspension was found to be 1.6 ± 0.2 and 3.38 ± 1.0, respectively. Taken together, a significant improvement in retinal morphological alterations was observed in the ST-CUBs treated group, which was notably higher than that in the diabetic group (*p* < 0.05) (Figure 7F).

#### 3.6.2. Immunohistochemistry (VEGF, Pro-Inflammatory Cytokines, and Adhesion Molecules)

**Figure 8:**
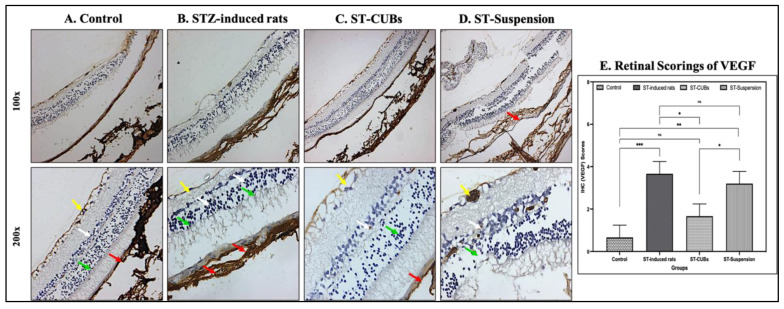
Immunohistostaining of VEGF in retina of rat (at 100× and 200×). (**A**) Control, (**B**) STZ-induced rats (2 months), (**C**) ST-CUB-treated STZ-induced rats, (**D**) ST-Suspension-treated STZ-induced rats. White arrow represents INL, green arrow represents ONL, yellow arrow represents GCL, and red arrow represents pigmented and choroid layers. (**E**) Unbiased mean scores of VEGF marker expression on IHC retinal sections at 100× and 200× by blinded spectators on different study groups where 0 signifies no VEGF expression and 4 signifies severe VEGF expression (mean ± SD, *n* = 3). The images present a generalized representation of the significant differences (* *p* ≤ 0.05, ** *p* ≤ 0.01, *** *p* ≤ 0.001) in SCJ administration between the ST-CUBs group and the ST-Suspension group. ^ns^ represents non-significant differences at *p* > 0.05.

The immunohistochemistry for VEGF in the retina of the control group showed normal immune reactivity against VEGF in the pigmented and choroid layers and no immune reactivity in rods and cones, INL, ONL, and GCL. The diabetic control/STZ-induced group showed severe immune reactivity against VEGF in the pigmented and choroid layers and moderate immune reactivity in rods and cones, INL (white arrow), ONL (green arrow), and GCL (yellow arrow). The ST-CUB-treated group showed mild immune reactivity in the pigmented and choroid layers (red arrow), rods and cones, INL, and ONL and moderate immune reactivity in GCL, whereas the ST-Suspension-treated group showed moderate immune reactivity in pigmented and choroid layers of the retina, rods and cones, INL, ONL, and GCL (Figure 8). The scoring of VEGF expression (in %) showed a significant rise in the induced diabetic group compared to other experimental groups (Figure 8E). In fact, these studies revealed that ST-CUBs can inhibit VEGF expression by hindering the activation of the transcriptional gene.

**Figure 9:**
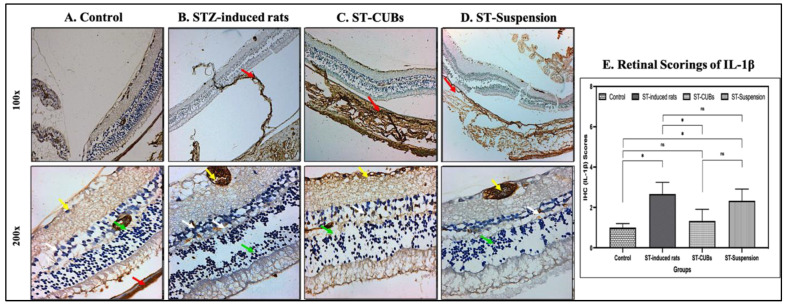
Immunohistostaining of IL-1β in retina of rat (at 100× and 200×). (**A**) Control, (**B**) STZ-induced rats (2 months), (**C**) ST-CUB-treated STZ-induced rats, (**D**) ST-Suspension-treated STZ-induced rats. White arrow represents INL, green arrow represents ONL, yellow arrow represents GCL, and red arrow represents pigmented and choroid layers. (**E**) Unbiased mean scores of IL-1β marker expression on IHC retinal sections at 100× and 200× by blinded spectators on different study groups where 0 signifies no IL-1β expression and 4 signifies severe IL-1β expression (mean ± SD, *n* = 3). The images provide a generalized depiction of how SCJ administration of ST-CUBs was significantly different (* *p* ≤ 0.05) in comparison to the STZ-induced group. ^ns^ represents non-significant at *p* > 0.05.

In control rats, IL-1β showed no immune reactivity in neuronal cells in the INL and neuronal cells, but mild immune reactivity was observed in pigmented and choroid layers of the retina, photoreceptor cells in the ONL, and rods and cones. Diabetic control rats exhibited moderate immune reactivity in the pigmented and choroid layers of the retina, while other layers of the retina showed very mild immune reactivity against IL-1β. The ST-CUB-treated group displayed normal immune reactivity in neuronal cells in the INL, photoreceptor cells in the ONL, and moderate immune reactivity in the GCL. In contrast, the ST-Suspension-treated group showed mild immune reactivity in neuronal cells in the INL, but moderate immune reactivity in photoreceptor cells in the ONL and GCL (Figure 9).

**Figure 10:**
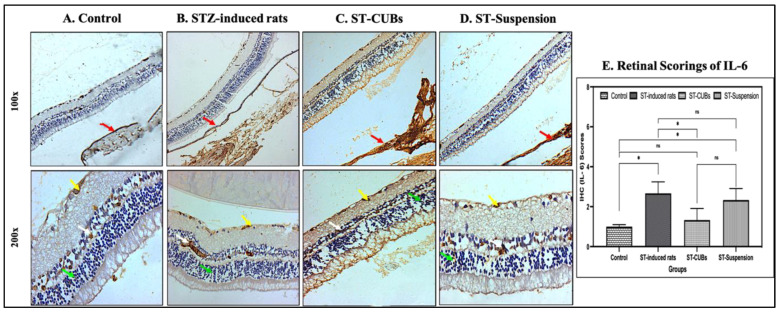
Immunohistostaining of IL-6 in retina of rat (at 100× and 200×). (**A**) Control, (**B**) STZ-induced rats (2 months), (**C**) ST-CUB-treated STZ-induced rats, (**D**) ST-Suspension-treated STZ-induced rats. White arrow represents INL, green arrow represents ONL, yellow arrow represents GCL, and red arrow represents pigmented and choroid layers. (**E**) Unbiased mean scores of IL-6 marker expression on IHC retinal sections at 100× and 200× by blinded spectators on different study groups where 0 signifies no IL-6 expression and 4 signifies severe IL-6 expression (mean ± SD, *n* = 3). The images provide a generalized depiction of how SCJ administration of ST-CUBs was significantly different (* *p* ≤ 0.05) in comparison to the STZ-induced group. ^ns^ represents non-significant at *p* > 0.05.

In the retina of the control group, IL-6 expression revealed no immune reactivity in neuronal cells in the INL, normal immune reactivity in neuronal cells and photoreceptor cells in the ONL, and mild immune reactivity in the GCL. The diabetic control group showed mild immune reactivity in neuronal cells and photoreceptor cells in the ONL. Moderate immune reactivity was noticed in the GCL, neuronal cells in the INL, and pigmented and choroid layers of the retina. The ST-CUB-treated group exhibited no immune reactivity against IL-6 in neuronal cells in the INL and mild immune reactivity in photoreceptor cells in the ONL, GCL, and pigmented and choroid layers of the retina. The ST-Suspension-treated group showed no immune reactivity in neuronal cells in the INL, moderate immune reactivity in neuronal cells and photoreceptor cells in the ONL, and mild immune reactivity in the GCL and pigmented and choroid layers of the retina (Figure 10).

Regarding the scoring of IL-1β and IL-6 expression (in %), there was a substantial increase in the induced diabetic group compared to other experimental groups (Figure 9E and Figure 10E). These results indicate a reduction in pro-inflammatory cytokine production, which could indicate a weaker association of these factors with ST-CUB treatment.

**Figure 11:**
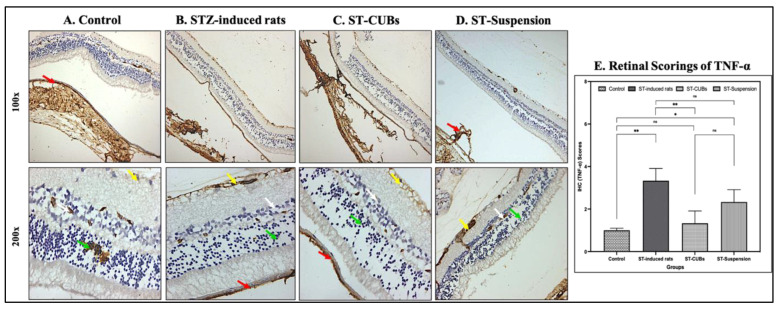
Immunohistostaining of TNF-α in retina of rat (at 100× and 200×). (**A**) Control, (**B**) STZ-induced rats (2 months), (**C**) ST-CUB-treated STZ-induced rats, (**D**) ST-Suspension-treated STZ-induced rats. White arrow represents INL, green arrow represents ONL, yellow arrow represents GCL, and red arrow represents pigmented and choroid layers. (**E**) Unbiased mean scores of TNF-α marker expression on IHC retinal sections at 100× and 200× by blinded spectators on different study groups where 0 signifies no TNF-α expression and 4 signifies severe TNF-α expression (mean ± SD, *n* = 3). The images provide a generalized depiction of how SCJ administration of ST-CUBs was significantly different (* *p* ≤ 0.05, ** *p* ≤ 0.01) in comparison to the STZ-induced group. ^ns^ represents non-significant at *p* > 0.05.

As for TNF-α in the retina, the control group exhibited normal immune reactivity in pigmented and choroid layers of the retina, ONL, and INL. The diabetic control group displayed moderate immune reactivity in pigmented and choroid layers, INL, ONL, and GCL. The ST-CUB treatment group exhibited normal immune reactivity in the pigmented and choroid layers of the retina, INL, ONL, and GCL, whereas the ST-Suspension group showed mild immune reactivity in the INL, ONL, and GCL against TNF-α (Figure 11).

With respect to the scoring of TNF-α expression (in %), there was a considerable increase in the induced diabetic group compared to the other experimental groups (Figure 11E). All these results indicated inhibited pleiotropic effects, which showed lower levels of TNF-α after treatment with ST-CUBs.

**Figure 12:**
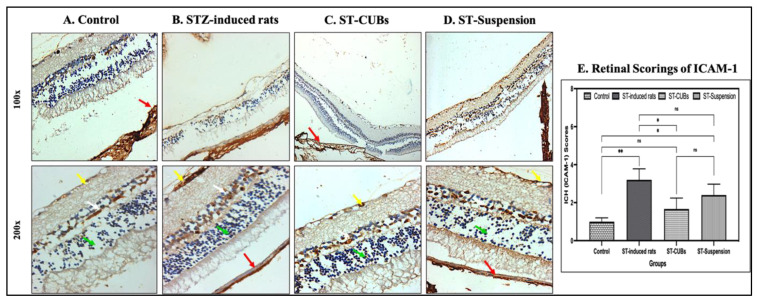
Immunohistostaining of ICAM-1 in retina of rat (at 100× and 200×). (**A**) Control, (**B**) STZ-induced rats (2 months), (**C**) ST-CUB-treated STZ-induced rats, (**D**) ST-Suspension-treated STZ-induced rats. White arrow represents INL, green arrow represents ONL, yellow arrow represents GCL, and red arrow represents pigmented and choroid layers. (**E**) Unbiased mean scores of ICAM-1marker expression on IHC retinal sections at 100× and 200× by blinded spectators on different study groups where 0 signifies no ICAM-1expression and 4 signifies severe ICAM-1expression (mean ± SD, *n* = 3). The images provide a generalized depiction of how SCJ administration of ST-CUBs was significantly different (* *p* ≤ 0.05 and ** *p* ≤ 0.01) in comparison to the STZ-induced group. ^ns^ represents non-significant at *p* > 0.05.

In retinal immunohistochemistry for ICAM-1, the control group showed normal immune reactivity in the pigmented and choroid layers of the retina, ONL, and INL, whereas the diabetic control group exhibited mild immune reactivity. Both the ST-CUB- and ST-Suspension-treated groups displayed normal immune reactivity in the ONL, INL, and GCL (Figure 12). The scoring of ICAM-1 expression (in %) revealed a significant increase in the induced diabetic group compared to the further experimental groups (Figure 12E). These results indicated reduced ICAM-1 expression in the retinal vasculature after treatment with ST-CUBs and ST-Suspension.

## 4. Discussion

Our research focuses on the development of a cubosome nanocarrier system prepared using a hot melt dispersion emulsification technique for subconjunctival delivery. To achieve effective ocular targeting, it is essential to maintain specific physical attributes such as particle size, polydispersity index (PDI), and zeta potential. The particle size of ST-CUBs ranged from 100 to 300 nm, with a narrow PDI, which is highly preferred for effective ocular targeting. This small particle size provides a larger surface area, resulting in an increased ST release rate. The small PDI indicates a monomodal dispersion of CUB particles, allowing for prolonged drug release through colloidal dispersion. Additionally, high drug entrapment efficiency is crucial for estimating drug concentration at the drug absorption site. HR-TEM analysis confirmed the cubic morphology in the nanometric range. The formation of CUBs was facilitated by the interface of GMO/F-127 with water at a specific temperature, aided by hydrogen bonds [38]. A higher % EE is always desirable as it leads to better drug release at the absorption site. Nanoparticle stability is a significant concern in terms of safety and efficacy. The stability of the colloidal dispersion system depends on the electrostatic repulsion between the lipid carriers dispersed in the CUBs, which further plays a role in the cell membrane interface. The prepared ST-CUBs were highly unstable at 2–8 °C but stable at room temperature (25 °C). Therefore, it is necessary to store the formulation at 25 °C to preserve its physicochemical parameters. These results and parameters align with existing literature [39,40]. Thus, the storage stability attributes of ST-CUBs are suitable for their use as a novel lipid nanocarrier for ocular drug delivery.

The fluorescence intensity in the posterior eye segment of subconjunctivally administered RhB dye solution indicates the ability of CUBs to easily cross barriers and distribute in posterior ocular tissues (retina/choroid and vitreous). The high fluorescence intensity in the posterior eye segment of subconjunctivally administered ST-CUBs can be attributed to the direct accessibility of dye encapsulated in the CUB formulation to the posterior tissue. The insertion of the formulation underneath the conjunctiva provides direct entry to the sclera, delivering a high drug concentration in the retina and vitreous humor via the transscleral route through paracellular transport for drug diffusion [41]. However, adsorptive endocytosis internalization of ST-CUBs and the potential of CUBs to access tight junctions and penetrate through paracellular transport when injected by the transscleral route can be assumed. Therefore, ST-CUBs are capable of efficiently transporting the desired drug concentration to the posterior tissues in the periocular space, particularly in the retina.

The subconjunctival route offers a promising and less-invasive periocular route for delivering therapeutics to the retina to manage various posterior ocular pathologies by providing direct entry to the transscleral route [42]. Safety is a primary concern with any novel dosage form. Visual inspection and gross pathology of rats remained unchanged even after 7 days of treatment via SCJ injection. Pharmacokinetic studies showed that the ST concentration in the vitreous humor and retina was significantly higher in the ST-CUB group than in the ST-suspension group. The higher drug concentration in the retina is crucial for the treatment of DR by controlling VEGF expression in diabetes. The higher concentration of ST in the ST-CUB group is due to the prolonged retention and residence time in the target tissue (retina), which is indeed due to the smaller size of CUBs. Therefore, the achieved concentration of ST in the retina will be sufficient to nullify the elevated concentration of VEGF in the retina.

The higher C_max_ of ST-CUBs may be due to the self-assembled crystalline structure of CUBs that incorporates the highly hydrophobic drug/ST and the better retention time in the posterior ocular cavity, which enhances the permeation property. The high T_max_ of ST from ST-CUBs is due to sustained release. ST encapsulation in CUBs significantly lowered the values of the elimination rate constant and prolonged the half-life compared to ST-Suspension. The increase in AUC for CUBs is likely due to greater dissolved-drug levels for the CUBs. All these factors are responsible for enhancing the ocular permeation of the formulation into the retina by circumventing the BRB, thereby improving the therapeutic efficacy of ST. Importantly, the in vivo findings suggest that SCJ administration opens the door for less-invasive drug delivery to the retina.

Diabetic rats developed early stages of retinopathy within a few months after the onset of hyperglycemia. The rat model has been commonly used for DR studies because it is easy to handle and cost-effective. Streptozotocin (STZ) is commonly used as a chemical inducer of diabetes in animal models that destroys pancreatic islet β cells and is a popular model for non-proliferative DR studies. STZ-induced diabetic rats exhibited common features of human NPDR such as degeneration of capillaries, dilation of the blood vessels, increased vascular permeability, increased leukostasis, microaneurysms, pericyte loss, vascular leakage, breakdown of the BRB, loss and damage of ganglion and endothelial cells, and thickening of the capillary basement membrane [43]. Preclinical studies reported the observation of elevated levels of VEGF and inflammatory cytokines in the retina of diabetic rats, similar to those noticed in diabetic patients [44]. ST-CUB treatment showed significant inhibition in retinal morphology changes compared to the ST-Suspension and diabetic groups, resembling the control group.

Immunohistochemistry studies concurrently detected changes in the expression of VEGF, IL-1β, IL-6, TNF-α, and ICAM-1 in the retinal sections of the rats in all experimental groups. The scoring of VEGF, IL-1β, IL-6, TNF-α, and ICAM-1 expression revealed a significant increase in the induced diabetic group compared to the control group and a significant reduction in the ST-CUB group compared to the ST-Suspension group. These results indicated the downregulation of VEGF and pro-inflammatory cytokine production, demonstrating the superior efficacy of ST-CUBs compared to the ST-Suspension and diabetic groups. Hence, the size of the nanoparticles significantly influenced the specific immune response, highlighting the importance of both size and shape in nanoparticle distribution. All these findings indicate a dominant effect of cubosome size on the immune response in the retinas of diabetic rats.

## 5. Conclusions

The distribution of dye-loaded ST-CUBs demonstrates the retention of nanocarriers in the posterior eye segment after SCJ administration. The significantly higher concentration of ST in the retina compared to the vitreous humor of the ST-CUB group under a single-dose pharmacokinetic study confirms sustained retinal delivery. ST-CUBs showed about a 5-fold increase in the AUC_0–24_ in the target tissue (retina) compared to ST-Suspension, enhancing drug bioavailability. IHC studies indicate a significant reduction in the expression of VEGF, cytokines, and adhesion molecules in the DR model after ST-CUBs treatment compared to ST-Suspension. In conclusion, ST-loaded CUBs show promise as an effective nanocarrier for overcoming biological barriers, potentially leading to the development of less-invasive therapies through the SCJ route. Further extensive safety and efficacy studies are recommended to translate this delivery model for clinical use.

## Figures and Tables

**Table 1:** Treatment outline for pharmacokinetic study.

Group	Substance	No. of Animals with Sample Points	Average Weight (g) (Mean ± SD)	Treatment
I	PBS (pH 7.4)	18 in each group and 3 at the same point	218.27 ± 9.96	A single SCJ injection of PBS pH 7.4, 50 µL
II	ST-CUBs	18 in each group and 3 at the same point	214.16 ± 5.76	A single SCJ injection of ST-loaded cubic nanocarriers, 50 µL
III	ST-Suspension	18 in each group and 3 at the same point	210.38 ± 4.11	A single SCJ injection of ST-Suspension, 50 µL

**Table 2:** Evaluation of PK parameters of ST-CUBs and ST-Suspension in plasma, vitreous humor, and retina of the treated left eye (mean ± SD, *n* = 3).

PK Parameters	Type of Formulation Administered to Left Eye of Rat					
	Plasma		Vitreous Humor		Retina/Choroid	
	ST-Suspension	ST-CUBs	ST-Suspension	ST-CUBs	ST-Suspension	ST-CUBs
C_max_ (ng/mL)	349.67 ± 48.63	101.65 ± 17.60 ****	46.83 ± 8.43	216.48 ± 21.72****	246.94 ± 43.14	820.75 ± 37.41 ****
T_max_ (h)	2	4	4	4	2	4
K_el_ (h^−1^)	0.0399 ± 0.031	0.0275 ± 0.003 *	0.0340 ± 0.0012	0.0336 ± 0.005 *	0.0561 ± 0.06	0.0420 ± 0.004 *
T_1/2_ (h)	17.339 ± 0.645	25.156 ± 0.612 ****	20.356 ± 0.864	20.579 ± 0.673 *	12.338 ± 0.283	16.4756 ± 0.215 ****
AUC_(0–24)_ (ng·h/mL)	2016.0 ± 68.71	739.49 ± 39.54 ****	332.05 ± 26.42	1679.93 ± 38.66 ****	1572.6 ± 46.51	7896.24 ± 101.31 ****
AUC_(24–∞)_ (ng·h/mL)	237.10 ± 56.46	239.12 ± 29.52 *	116.8699 ± 80.1	1115.99 ± 43.42 ****	110.93 ± 91.27	2560.7 ± 45.66 ****
AUC_(0–∞)_ (ng·h/mL)	2253.1 ± 65.74	978.61 ± 32.09 ***	448.92 ± 101.32	2795.92 ± 85.31 ****	1683.5 ± 89.72	10,457.0 ± 189.63 ****
AUMC_(0–24)_ (ng·h^2^/mL)	11,869.1 ± 42.12	5177.9 ± 74.89 ****	2363.7 ± 61.54	14,746.66 ± 86.12 ****	9082.3 ± 55.8	61,660.7 ± 92.11 ****
AUMC_(24–∞)_ (ng·h^2^/mL)	11,623.2 ± 62.31	14,419.2 ± 94.3 ****	6237.92 ± 55.8	59,924.4 ± 43.9 ****	4637.4 ± 39.08	122,339.7 ± 60.1 ****
AUMC_(0–∞)_ (ng·h^2^/mL)	23,492.4 ± 90.98	19,597.2 ± 76.51 ****	8601.6 ± 68.9	74,671.13 ± 62.3 ****	13,719.8 ± 88.5	184,000.5 ± 81.69 ****
MRT_(0–∞)_ (h)	10.4262 ± 47.8	20.025 ± 39.4 **	19.1607 ± 45.3	26.707 ± 53.08 **	8.1494 ± 54.3	17.595 ± 52.96 **
RB (Retina) (%)	502.11 ± 10.24					

Data represented as mean ± SD. Significance was calculated using unpaired *t*-test. The PK parameters of ST-CUBs were significantly different from those of ST-Suspension at * *p* ≤ 0.05, ** *p* ≤0.01, *** *p* ≤0.001, and **** *p* ≤ 0.0001.

## Data Availability

The authors confirm that the data supporting the findings of this study are available within the article. Additional data will be available on request.
